# Changes in Parental Attitudes Toward COVID-19 Vaccination and Routine Childhood Vaccination During the COVID-19 Pandemic: Repeated Cross-sectional Survey Study

**DOI:** 10.2196/33235

**Published:** 2022-05-13

**Authors:** Qiang Wang, Shixin Xiu, Liuqing Yang, Ying Han, Tingting Cui, Naiyang Shi, Minqi Liu, Youqin Yi, Chang Liu, Xuwen Wang, Guoping Yang, Lili Ji, Weijie Zhou, Hui Jin, Shiqi Zhen, Leesa Lin

**Affiliations:** 1 Department of Epidemiology and Health Statistics School of Public Health Southeast University Nanjing China; 2 Key Laboratory of Environmental Medicine Engineering, Ministry of Education School of Public Health Southeast University Nanjing China; 3 Wuxi Center for Disease Control and Prevention Wuxi China; 4 Jiangsu Provincial Center for Disease Control and Prevention Nanjing China; 5 Department of Infectious Disease Epidemiology London School of Hygiene and Tropical Medicine London United Kingdom; 6 Laboratory of Data Discovery for Health Hong Kong Science Park Hong Kong China

**Keywords:** childhood vaccination, COVID-19 vaccine, vaccine hesitancy, repeated cross-section survey

## Abstract

**Background:**

It was reported that one in four parents were hesitant about vaccinating their children in China. Previous studies have revealed a declining trend in the vaccine willingness rate in China. There is a need to monitor the level of parental vaccine hesitancy toward routine childhood vaccination and hesitancy toward the COVID-19 vaccine during the ongoing COVID-19 pandemic.

**Objective:**

This study aims to assess changes in trends of parental attitudes toward routine childhood vaccines and COVID-19 vaccinations across different time periods in China.

**Methods:**

Three waves of cross-sectional surveys were conducted on parents residing in Wuxi City in Jiangsu Province, China from September to October 2020, February to March 2021, and May to June 2021. Participants were recruited from immunization clinics. Chi-square tests were used to compare the results of the three surveys, controlling for sociodemographic factors. Binary and multivariable logistic regression analysis was used to examine factors related to parental vaccine hesitancy and COVID-19 vaccine willingness.

**Results:**

Overall, 2881, 1038, and 1183 participants were included in the survey’s three waves. Using the Vaccine Hesitancy Scale, 7.8% (225/2881), 15.1% (157/1038), and 5.5% (65/1183) of parents showed hesitancy to childhood vaccination (*P*<.001), and 59.3% (1709/2881), 64.6% (671/1038), and 92% (1088/1183) of parents agreed to receive a COVID-19 vaccine themselves in the first, second, and third surveys, respectively (*P*<.001). In all three surveys, “concerns about vaccine safety and side effects” was the most common reason for refusal.

**Conclusions:**

There has been an increasing acceptance of COVID-19 vaccination in Wuxi City, China. Effective interventions are needed to mitigate public concerns about vaccine safety.

## Introduction

Vaccination is considered one of the most successful interventions in disease prevention. Annually, it prevents 2 to 3 million deaths from vaccine-preventable diseases (VPDs), including diphtheria, tetanus, pertussis, influenza, and measles [1]. However, vaccine hesitancy, which refers to delaying or refusing vaccines, threatens the success of vaccination and is among the most important current global health concerns [2,3]. Parental hesitancy to childhood vaccines can decrease vaccination coverage among children; moreover, numerous VPDs continue to spread because of low vaccine uptake. For example, the Asia-Pacific region reported 63,483 pertussis cases in 2019 [4], and EU countries reported 148,279 measles cases from 2010 to 2019 [5].

Surveys on parental childhood vaccine hesitancy have been conducted globally since 2011 [6]. According to a national survey in the United States, one in five parents showed hesitation toward childhood vaccinations from 2018 to 2019 [7]. A 2018 survey of 5736 samples conducted in 18 European countries showed that the hesitancy rate among parents ranged from 9% (Portugal) to 42% (Israel) [8]. A 2020 survey conducted in Peru reported a vaccine hesitancy rate among parents of approximately 10% [9].

In China in 2017, VPDs were reported in 280,315 children and adolescents [10]. In addition, a study conducted in 2015 in Zhejiang Province, China reported that one in four parents were hesitant about vaccinating their children [11]. This data suggests a need to address parental vaccine hesitancy toward routine childhood vaccines in China. Specifically, there is a need to monitor both children’s immunization coverage and the level of parental vaccine hesitancy.

During the ongoing COVID-19 pandemic, hesitancy toward the COVID-19 vaccine is a substantial concern. Subsequently, there have been worldwide surveys on the acceptability of COVID-19 vaccines, revealing widely varying levels of acceptability across countries [12,13]. A meta-analysis of 38 studies including 81,173 individuals showed that the acceptance rate ranged from 94.31% (Malaysia) to 43.38% (Greece) [13].

Since March 2020, numerous Chinese studies have been conducted on COVID-19 vaccination willingness [14-18]. These data demonstrate that the willingness rate in China varied between 52.2% and 83.8%, and that the changing trend in willingness rates warrants monitoring. One repeated cross-sectional study and two longitudinal studies have revealed a declining trend in the vaccine willingness rate in China [19-21]; this could substantially impede efforts to contain COVID-19, especially with the rise of Delta and other variants. However, most of these studies were performed before the COVID-19 vaccine rollout in China [14-21]. A cohort study conducted in the United States reported increased vaccine acceptability after the vaccination program commenced [22]. Therefore, the acceptability of vaccines needs to be reassessed in China, especially after the COVID-19 vaccine’s rollout. As of August 18, the cumulative number of COVID-19 vaccines administered in mainland China exceeded 1.9 billion [23]. Furthermore, vaccine policies and strategies in China have evolved, bolstering the need to monitor public reactions toward COVID-19 vaccination regularly.

Our study aimed to assess changes in the level of parental vaccine hesitancy toward routine childhood vaccines and public acceptance of COVID-19 vaccines at different times in China, especially after their rollout. Specifically, we aimed to examine the reasons for accepting or refusing the COVID-19 vaccine across various time intervals. In addition, we examined changes in both the number of administered COVID-19 vaccine doses and the vaccination strategies in the first half of 2021 to assess actual vaccination decisions in Wuxi City in Jiangsu Province.

## Methods

### Study Design and Participants

We conducted three waves of cross-sectional surveys in Wuxi City (total population: 6.59 million in 2018), located in Eastern China, from September 21 to October 17, 2020; February 9 to March 13, 2021; and May 24 to June 10, 2021. The three surveyed periods corresponded with three stages of COVID-19 vaccine development and rollout: COVID-19 vaccine trials (first survey), before mass COVID-19 vaccination (second survey), and during mass COVID-19 vaccination (third survey). We recruited participants from 6 immunization clinics across the city. The selection method of vaccination clinics has been previously described by Wang et al [24]. The sample size was calculated as 
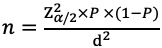
; 90% COVID-19 vaccination willingness rate (P) [14], 2.5% precision (d), and 5% type I error (α); the final size was 959 participants.

An informed consent form and a self-administered questionnaire were distributed to the parents of all children treated at the selected vaccination clinics during the survey periods. Parents were informed about the study purpose and anonymization of the investigation. Paper-form questionnaires were used during the first survey period, while online questionnaires were used during the second and third survey periods. The online questionnaire was created and distributed through the Wenjuanxing website. The participants accessed and completed the questionnaire by scanning a QR code (2D barcode). All potential participants were assured that participation in the research was voluntary and that they would be free to discontinue participation at any time.

The inclusion criteria included the father or mother being with the child (aged ≤6 years), and when both parents visited the clinic simultaneously, the one who self-identified as the child’s primary caregiver completed the questionnaire. The exclusion criteria included the father or mother being younger than 18 years and parents having mental illnesses.

### Ethics Approval

Wuxi Center for Disease Control and Prevention Ethics Committee approved the surveys (2020No10).

### Measures and Data Collection

The questionnaire comprised three parts: sociodemographic characteristics, parental vaccine hesitancy, and willingness to receive COVID-19 vaccination. The first survey comprised questions regarding sociodemographic characteristics, including the participant’s age, sex, educational level, annual household income, and health care occupation status. The subsequent surveys added four additional questions regarding the number of people in residence, contacts per day, self-reported health, and influenza vaccination status in the last season. These questions were all specific to participants. The questions regarding parental vaccine hesitancy toward routine childhood vaccines referred to the 10-item Vaccine Hesitancy Scale (VHS) developed by the Strategic Advisory Group of Experts [25]. The 10-item VHS has been used in numerous countries with acceptable reliability and validity [26-28]. We used a 5-point scale (strongly disagree: 1; disagree: 2; neither agree nor disagree: 3; agree: 4; or strongly agree: 5) for responses to each VHS item.

During the vaccine trial period, one question, “If the COVID-19 vaccine was available, will you vaccinate yourself?” was used to measure the participants’ willingness to accept a COVID-19 vaccination (responses: “yes,” “not sure,” and “no”). The next question asked for specific reasons for acceptance or refusal (If “yes,” “why?” or if “no/not sure,” “why?”). The other two surveys replaced this question with “Will you vaccinate against COVID-19 for yourself?” as the COVID-19 vaccine had become available in China in January 2021. Other options were also added to the survey for answers regarding the reasons for accepting or refusing a COVID-19 vaccination. These questionnaires are provided in Multimedia Appendix 1.

COVID-19 vaccination records were derived from the information management system for COVID-19 vaccines to assess actual vaccination decisions. Furthermore, governmental vaccination strategies were obtained from the official websites of relevant health authorities (Multimedia Appendix 2).

### Statistical Analysis

All analyses were performed using R software (R Foundation for Statistical Computing). Categorical variables are expressed using frequencies and percentages while continuous variables are presented as means and SDs.

We calculated the VHS score using the participants’ responses to the 10 items [24], with a lower score indicating a higher hesitancy level. Parental vaccine hesitancy to routine childhood vaccines was classified as either low or high hesitancy (VHS score>30 and ≤30, respectively). Regarding the analyses of COVID-19 vaccination willingness, “no” and “not sure” responses were combined into a “refusal” response. Samples from the second and third surveys were directly standardized according to the age, gender, and medical occupation status distribution of the sample from the first survey to ensure comparability of the findings across all surveys [19]. Intersurvey comparisons were performed using the chi-square or Fisher exact test. A two-sided *P* value <.05 was considered statistically significant. Pairwise comparisons among groups were performed with Bonferroni correction.

Binary logistic regression analysis was used to examine factors related to parental vaccine hesitancy and COVID-19 vaccine willingness. Outcome variables included parental vaccine hesitancy and COVID-19 vaccine willingness. Independent variables included sex, age, educational level, annual household income, health care occupation status, number of people in residence, number of contacts per day, self-reported health, and influenza vaccination status in the last season. Regression analyses included data from the second and third surveys as some important variables (including influenza vaccination experience) were not queried in the first survey. The variables with *P*<.10 in the univariate regression model were included in the multivariable regression model. A 95% CI for the crude odds ratio was derived from univariate analysis. A 95% CI for the adjusted odds ratios (AORs) was derived from multivariable analyses. A two-sided *P*<.05 in the multivariable analyses was considered significant.

## Results

### Sociodemographic Characteristics of the Participants

Overall, 2881 (response rate 79.9%), 1038 (response rate 78.7%), and 1183 (response rate 79.3%) participants were included in the first, second, and third surveys, respectively (Table 1). The average ages of the responders in the first, second, and third surveys were 31.36 (SD 4.38), 33.36 (SD 4.74), and 32.12 (SD 5.49) years, respectively. In the first, second, and third surveys, 69.5% (2001/2881), 89.1% (925/1038), and 82.9% (980/1183) of participants, respectively, had an education level of college (or equivalent) or above. Additionally, 22.1% (229/1038) and 20.9% (247/1183) of participants in the second and third surveys, respectively, reported receiving an influenza vaccination in the last season.

**Table 1:** Participant’s sociodemographics in three cross-section surveys.

Variables		COVID-19 vaccine trials period (September to October 2020; n=2881)	Premass COVID-19 vaccination period (February to March 2021; n=1038)	Ongoing mass COVID-19 vaccination period (May to June 2021; n=1183)
**Sex, n (%)**				
	Women	2146 (74.5)	699 (67.3)	680 (57.5)
	Men	735 (25.5)	339 (32.7)	503 (42.5)
Age (years), mean (SD)		31.36 (4.38)	33.36 (4.74)	32.12 (5.49)
**Age group (years), n (%)**				
	<26	248 (8.6)	32 (3.1)	116 (9.8)
	26-30	1086 (37.7)	239 (23)	365 (30.9)
	31-35	1112 (38.6)	475 (45.8)	418 (35.3)
	36-40	356 (12.4)	216 (20.8)	201 (17.0)
	≥41	79 (2.7)	76 (7.3)	83 (7.0)
**Educational level, n (%)**				
	Junior high school or below	338 (11.7)	21 (2.0)	40 (3.4)
	High school graduate or equivalent	542 (18.8)	92 (8.9)	163 (13.8)
	College or equivalent	1791 (62.2)	755 (72.7)	880 (74.4)
	Master’s diploma or above	210 (7.3)	170 (16.4)	100 (8.5)
**Annual household income (RMB; US $), n (%)**				
	<50,000 (<7669)	206 (7.2)	53 (5.1)	79 (6.7)
	50,000 to <100,000 (7669 to <15,337)	850 (29.5)	264 (25.4)	348 (29.4)
	100,000 to <150,000 (15,337 to <23,006)	754 (26.2)	277 (26.7)	304 (25.7)
	≥150,000 (≥23,006)	1071 (37.2)	444 (42.8)	452 (38.2)
**Health care occupation, n (%)**				
	Yes	181 (6.3)	449 (43.3)	287 (24.3)
	No	2700 (93.7)	589 (56.7)	896 (75.7)
**Number of people in residence, n (%)**				
	1	—^a^	31 (3.0)	26 (2.2)
	2-5	—	902 (86.9)	1003 (84.8)
	≥6	—	105 (10.1)	154 (13.0)
**Number of contacts per day, n (%)**				
	1-10	—	544 (52.4)	544 (45.2)
	11-20	—	251 (24.2)	251 (28.2)
	≥21	—	243 (23.4)	243 (26.5)
**Self-reported health, n (%)**				
	Very good	—	378 (36.4)	204 (17.2)
	Good	—	507 (48.8)	517 (43.7)
	Fair	—	150 (14.5)	420 (35.5)
	Poor	—	1 (0.1)	28 (2.4)
	Very poor	—	2 (0.2)	14 (1.2)
**Influenza vaccination in the last season, n (%)**				
	No	—	809 (77.9)	936 (79.1)
	Yes	—	229 (22.1)	247 (20.9)

^a^These items were not queried about in the first questionnaire.

### Parental Vaccine Hesitancy and COVID-19 Vaccination Willingness

In Multimedia Appendix 3 and Multimedia Appendix 4, Figure S1, the rate of high hesitancy toward childhood vaccines was 7.8% (225/2881), 17.8% (157/1038), and 5.5% (65/1183) in the COVID-19 vaccine trial, premass COVID-19 vaccination, and ongoing mass COVID-19 vaccination periods, respectively. The COVID-19 vaccination willingness was 59.3% (1709/2881), 64.6% (671/1038), and 92% (1088/1183) in the COVID-19 vaccine trial, premass COVID-19 vaccination, and ongoing mass COVID-19 vaccination periods, respectively. The willingness rate continuously increased and was the highest in the third survey. There were significant intersurvey differences in the “high hesitancy toward childhood vaccination” rate and COVID-19 vaccination willingness (*P*<.001 and *P*<.001, respectively).

### Administered COVID-19 Vaccine Doses in Wuxi City

As shown in Multimedia Appendix 4, Figure S2, the cumulative number of administered COVID-19 vaccines in Wuxi City exceeded 10 million doses by July 2021. The vaccination strategy varied over time. During the early period (between January and March), a select population was vaccinated against COVID-19. From June, vaccines were administered to people 18 years and older.

### Factors Associated With Parental Vaccine Hesitancy

Sex and self-reported health status were associated with parental vaccine hesitancy (Table 2 and Multimedia Appendix 4, Figure S3). Compared with women, men were more likely to show hesitancy (AOR 1.372, 95% CI 1.028-1.832). Compared with participants who reported having very good health, those who reported only good health were less likely to be hesitant about childhood vaccines (AOR 0.549, 95% CI 0.399-0.755).

**Table 2:** Univariable factors associated with parental vaccine hesitancy to routine childhood vaccine and COVID-19 vaccine willingness.

Variables		Parental vaccine hesitancy to routine childhood vaccine^a^			COVID-19 vaccine willingness^b^	
		COR^c^ (95% CI)	*P* value	COR (95% CI)		*P* value
**Sex (female as reference)**						
	Male	1.260 (0.959-1.654)	.10	1.906 (1.498-2.425)		<.001
**Age group (years; <26** **as reference)**						
	26-30	0.995 (0.558-1.775)	.99	0.543 (0.295-1.000)		.05
	31-35	0.834 (0.473-1.470)	.53	0.356 (0.197-0.644)		.001
	36-40	1.253 (0.694-2.264)	.45	0.382 (0.206-0.708)		.002
	≥41	1.187 (0.590-2.389)	.63	0.516 (0.253-1.051)		.07
**Educational level (junior high school or below as reference)**						
	High school graduate or equivalent	1.493 (0.554-4.023)	.43	1.373 (0.558-3.379)		.49
	College or equivalent	1.266 (0.500-3.204)	.62	0.579 (0.261-1.286)		.18
	Master’s diploma or above	1.779 (0.669-4.731)	.25	0.337 (0.147-0.774)		.01
**Annual household income (RMB; US $; <50,000 [<7669] as reference)**						
	50,000 to <100,000 (7669 to <15,337)	0.888 (0.504-1.564)	.68	1.383 (0.833-2.296)		.21
	100,000 to <150,000 (15,337 to <23,006)	0.693 (0.388-1.239)	.22	1.053 (0.639-1.736)		.84
	≥150,000 (≥23,006)	0.812 (0.468-1.409)	.46	0.725 (0.45-1.167)		.19
**Health care occupation (no as reference)**						
	Yes	1.447 (1.099-1.905)	.008	1.262 (0.997-1.598)		.05
**Number of people in residence (1 as reference)**						
	2-5	0.545 (0.271-1.096)	.09	1.059 (0.543-2.065)		.87
	≥6	0.616 (0.282-1.345)	.22	1.271 (0.608-2.659)		.52
**Number of contacts per day (1-10 as reference)**						
	11-20	0.764 (0.548-1.066)	.11	1.397 (1.073-1.819)		.01
	≥21	0.791 (0.565-1.106)	.17	1.664 (1.257-2.202)		<.001
**Self-reported health (very good as reference)**						
	Good	0.453 (0.332-0.617)	<.001	0.724 (0.550-0.954)		.02
	Fair	0.454 (0.315-0.655)	<.001	0.778 (0.570-1.062)		.11
	Poor	0.790 (0.269-2.321)	.67	0.461 (0.198-1.075)		.07
	Very poor	0.706 (0.158-3.154)	.65	0.762 (0.213-2.728)		.68
**Influenza vaccination in the last season (no as reference)**						
	Yes	0.918 (0.657-1.282)	.62	5.764 (3.702-8.974)		<.001
**Survey (second survey as reference)**						
	Third survey	0.304 (0.226-0.409)	<.001	6.118 (4.712-7.944)		<.001

^a^For parental vaccine hesitancy, “high-hesitant” was used as the reference.

^b^For COVID-19 vaccination willingness, “yes” was used as the reference.

^c^COR: crude odds ratio.

### Factors Associated With COVID-19 Vaccination Willingness

Table 2 and Multimedia Appendix 4, Figure S3 show that sex, educational level, participants’ health care occupation status, number of contacts per day, self-reported health status, and influenza vaccination history were associated with COVID-19 vaccination willingness. Participants in health care occupations were more likely to accept COVID-19 vaccinations (AOR 1.853, 95% CI 1.397-2.457). Compared with participants who reported that they were in very good health, those who reported good, fair, poor, or very poor health were more likely to refuse COVID-19 vaccination. Influenza vaccination in the last season was positively associated with willingness to receive COVID-19 vaccination (AOR 5.564, 95% CI 3.372-8.531).

### Reasons for Accepting or Refusing Vaccination Against COVID-19

In all three surveys, “Protect all the people you are around” was the most frequent reason stated for accepting the COVID-19 vaccine (Multimedia Appendix 4, Figure S4). Further, in all three surveys, “Concern about vaccine safety and side effects” was the most frequent reason for refusing COVID-19 vaccination. The second most frequent reasons for refusing COVID-19 vaccination were “doubt the vaccine effectiveness,” “no professional gave me a detailed introduction to the vaccine,” and “vaccination contraindications” in the first, second, and third surveys, respectively.

## Discussion

### Principal Findings

Our findings demonstrate that public attitudes toward routine childhood vaccines and the COVID-19 vaccine specifically varied across time. One in seven parents showed hesitancy toward routine childhood vaccines between February and March 2021. COVID-19 vaccination willingness showed a significantly increasing trend in Wuxi, China, from 59.3% to 92% (*P*<.001). In all three surveys, the most common reasons for parents’ accepting and refusing COVID-19 vaccines for themselves were “protecting all the people you are around” and “concern about vaccine’s safety and side effects,” respectively.

COVID-19 vaccine acceptability (>90%) was higher during the ongoing mass COVID-19 vaccination period than seen in other studies (varied between 52.2% and 83.8%) [14-18]. Moreover, the reported values were higher than those in most countries worldwide [13,29]. The vaccination willingness rate was estimated as 80.3% (95% CI 74.9%-85.6%) across low- and middle-income countries [29]. Consistent with previous findings [22], there was an upward trend (*P*<.001) in COVID-19 vaccine acceptability in Wuxi City, especially after the vaccine rollout. The willingness rate in the United States was estimated to increase from 54% to 65% between October 2020 and March 2021 [22]. However, one cohort study in England and Wales showed that the willingness rate decreased from 56% to 52% between December 2020 and February 2021 [30].

The cumulative number of administered COVID-19 vaccines to adults in Wuxi City exceeded 10 million doses by July 2021. A series of national and local interventions have been implemented to improve public acceptance of the COVID-19 vaccine. Specifically, the Chinese government has organized numerous press conferences to clarify the efficacy, safety, and importance of COVID-19 vaccines [31,32]. In addition, the attitudes and practices toward COVID-19 vaccination of China’s top public health influencers, including Dr Nanshan Zhong, a nationally famous scientist, were widely referred to as part of vaccine communications [33]. Local governments also produced slogans and short videos to promote vaccine acceptance [32].

Sex, education attainment, participants’ health care occupation status, number of contacts per day, self-reported health status, and influenza vaccination history were associated with parents’ COVID-19 vaccination acceptance for themselves. Health care workers (HCWs) constitute an important population, and HCWs have a higher risk of COVID-19 infection [34]. Moreover, HCWs are crucially involved in vaccination recommendations and administration [35-37]. Consistent with the findings from the systematic review, influenza vaccination in the last season was a strong positive predictor of COVID-19 vaccination [13]. The number of people in residence was not associated with parental vaccine hesitancy and COVID-19 vaccination willingness. Some participants, who tended to belong to single-parent families or divorced families, lived alone. Their child might live with their grandparents instead of their parents because a single father or mother could not care for their child due to work. Because of the necessity of signing informed consent before a child’s vaccination and grandparents who were not literate, the father or mother would accompany the child to clinics for vaccinations.

Consistent with previous studies [12,13,29], the most common reasons for refusal were concerns about safety and side effects. A systematic review reported that the rate of adverse events after COVID-19 vaccination was close to that of other routine vaccines [38]. The allergic reaction rate was approximately 2 cases per million doses for inactivated vaccines. For RNA vaccines, the rate of allergic reactions was approximately 2 to 5 cases per million doses [38]. There is a need to educate the public on the safety of the COVID-19 vaccine. Moreover, emerging SARS-CoV-2 variants have posed a threat to global immunity recently. COVID-19 breakthrough infections have been reported in vaccine recipients [39]. The emergence of breakthrough infections could cause public distrust in the COVID-19 vaccine. Surveillance of vaccine confidence regarding the influence of breakthrough infection events should be rapidly performed to allow specific responses to public concerns.

Additionally, to our knowledge, this is the first repeated cross-sectional study to assess changes in parental vaccine hesitancy toward routine childhood vaccines. There were significant intersurvey differences with large fluctuations; the hesitancy rate was the highest in the second survey (between February and March 2021). Our data identified a sudden increase in parental hesitancy toward routine childhood vaccines between February and March, immediately prior to the introduction of the mass COVID-19 vaccination policy.

### Limitations

Our study has several limitations. First, our choice of study design and sampling method might impede the generalizability of our findings as the surveys were performed in vaccination clinics in Wuxi City. In China, children must uptake a series of mandatory vaccinations before school entry [26]. Children who did not receive all of these vaccines were not allowed to go to school [26]. Hence, parents need to bring their children to the immunization clinics. We believe the representation of participants recruited from immunization clinics might be acceptable. However, the surveying in immunization clinics was still likely to cause a selection bias. Meanwhile, the self-selection bias in the surveys could not be ignored because parents showing concerns about vaccines were not likely to respond and complete the questionnaires. These parents might be more hesitant about childhood vaccinations. There is a need for a more rigorous study design (cohort study) and representative populations to provide more robust evidence. Second, findings regarding intersurvey comparisons should be interpreted cautiously because of differences in sociodemographic characteristics. To ensure intersurvey comparability of the results, we applied direct standardization. However, there were other factors that were not adjusted in the standardization, such as influenza vaccination history, that may produce a bias. However, we believe that these unstandardized factors would not influence the results significantly because the distributions of demographic characteristics in different surveys was approximated. Third, responses to questionnaires might be affected by complex factors, including recall bias and social desirability bias. Some factors associated with parental vaccine hesitancy, including marital status and child’s age, need to be explored further. Fourth, the methods for completing the questionnaire (via paper or the internet) differed across the surveys, leading to different responses. Fifth, we did not determine the causal relationship between vaccine hesitancy and health authority policies. More efforts should be made in further studies to investigate this link.

### Conclusion

In Wuxi City, China, three cross-sectional surveys revealed that 1 in 7 parents showed hesitancy toward routine childhood vaccines between February and March 2021. The acceptability of COVID-19 vaccines showed an increasing trend, especially after they became available (>90%). The cumulative number of administered COVID-19 vaccines to adults in Wuxi City has exceeded 10 million doses by July 2021. In all three survey waves, “concerns about vaccine safety and side effects” were the most common reason for refusal. Effective interventions need to be taken to mitigate public concerns about vaccine safety.

## Appendix

Multimedia Appendix 1 Questionnaires.

Multimedia Appendix 2 Official websites of relevant health authorities.

Multimedia Appendix 3 Participants’ vaccine hesitancy and COVID-19 vaccination willingness in three cross-section studies.

Multimedia Appendix 4 Supplementary figures.

## Abbreviations

AOR: adjusted odds ratio
HCW: health care worker
VHS: Vaccine Hesitancy Scale
VPD: vaccine-preventable disease
