# Supplementary material for: Changes in Parental Attitudes Toward COVID-19 Vaccination and Routine Childhood Vaccination During the COVID-19 Pandemic: Repeated Cross-sectional Survey Study
Source: JMIR Public Health Surveill. 2022 May 13;8(5):e33235. doi: 10.2196/33235 (PMC9109779; doi:10.2196/33235)
Supplement: Multimedia Appendix 1 [file publichealth_v8i5e33235_app1.docx]

**Attitudes to childhood vaccine and COVID-19 vaccine survey (questionnaire used in the first survey)**

1. **Demographics**

**A1. Birth：**_________year_________month

**A2. Sex：**□ Male □ Female

**A3. What’s your highest education level?**

□ Junior high school or below □ High school graduate or equivalent

□ College or equivalent □ Master's diploma or above

**A4. What’s your annual household income?**

□ < 50,000 RMB □ 50,000 RMB to < 100,000 RMB

□ 100,000 RMB to < 150,000 RMB □ ≥ 150,000 RMB

**A5. Is your job related to healthcare?**

□ Yes □ No

1. **Childhood vaccine attitudes**

**B1. Childhood vaccines are important for my child’s health**

□ Strongly disagree □ Disagree □ Neither agree nor disagree

□ Agree □ Strongly agree

**B2. Childhood vaccines are effective**

□ Strongly disagree □ Disagree □ Neither agree nor disagree

□ Agree □ Strongly agree

**B3. Having my child vaccinated is important for the health of others in my community**

□ Strongly disagree □ Disagree □ Neither agree nor disagree

□ Agree □ Strongly agree

**B4. All childhood vaccines offered by the government programme in my community are beneficial**

□ Strongly disagree □ Disagree □ Neither agree nor disagree

□ Agree □ Strongly agree

**B5. New vaccines carry more risks than older vaccines**

□ Strongly disagree □ Disagree □ Neither agree nor disagree

□ Agree □ Strongly agree

**B6. The information I receive about vaccines from the vaccine program is reliable and trustworthy**

□ Strongly disagree □ Disagree □ Neither agree nor disagree

□ Agree □ Strongly agree

**B7. Getting vaccines is a good way to protect my child/children from disease**

□ Strongly disagree □ Disagree □ Neither agree nor disagree

□ Agree □ Strongly agree

**B8. Generally I do what my doctor or health care provider recommends about vaccines for my child/children**

□ Strongly disagree □ Disagree □ Neither agree nor disagree

□ Agree □ Strongly agree

**B9. I am concerned about serious adverse effects of vaccines**

□ Strongly disagree □ Disagree □ Neither agree nor disagree

□ Agree □ Strongly agree

**B10. My child/children does or do not need vaccines for diseases that are not common anymore**

□ Strongly disagree □ Disagree □ Neither agree nor disagree

□ Agree □ Strongly agree

**C. COVID-19 vaccine attitudes**

**C1. If COVID-19 vaccine was available, will you vaccinate yourself?**

□ Yes (turn to C2) □ Not sure (turn to C3) □ No (turn to C3)

**C2. If “yes”, why? (Multiple choices)**

□ Risk of infection with COVID-19

□ COVID-19 is a serious disease

□ Protect the people around

□ COVID-19 vaccine is effective

□ Government publicity

□ Doctor's recommendation

□ Others

**C3. If “no/not sure”, why? (Multiple choices)**

□ COVID-19 is not serious

□ No risk of infection with COVID-19

□ Concern about side effects

□ Doubt the vaccine effectiveness

□ I don't have time

□ Generally reluctant to vaccinate

□ Fear needles

□ Worry about prices

□ Cannot acquire the vaccine

□ Want to immunize in a natural way rather than using a vaccine

□ Others

**Attitudes to childhood vaccine and COVID-19 vaccine survey (questionnaire used in the second and third survey)**

1. **Demographics**

**A1. Birth：**_________year_________month

**A2. Sex：**□ Male □ Female

**A3. What’s your highest education level?**

□ Junior high school or below □ High school graduate or equivalent

□ College or equivalent □ Master's diploma or above

**A4. What’s your annual household income?**

□ < 50,000 RMB □ 50,000 RMB to < 100,000 RMB

□ 100,000 RMB to < 150,000 RMB □ ≥ 150,000 RMB

**A5. Is your job related to healthcare?**

□ Yes □ No

**A6. How many people live in your current residence, including yourself?**

□ 1 □ 2 □ 3 □ 4 □ 5 □ 6 □ 7 □ 8 □ 9 □ 10 □ >10

**A7. How many people do you contact every day?**

□ 1-5 □ 6-10 □ 11-15 □ 16-20

□ 21-25 □ 26-30 □ 31-35 □ >35

**A8. How you rate your health?**

□ Very poor □ Poor □ Fair □ Good □ Very good

**A9. Have you had a flu shot in the last season?**

□ Yes □ No

1. **Childhood vaccine attitudes**

**B1. Childhood vaccines are important for my child’s health**

□ Strongly disagree □ Disagree □ Neither agree nor disagree

□ Agree □ Strongly agree

**B2. Childhood vaccines are effective**

□ Strongly disagree □ Disagree □ Neither agree nor disagree

□ Agree □ Strongly agree

**B3. Having my child vaccinated is important for the health of others in my community**

□ Strongly disagree □ Disagree □ Neither agree nor disagree

□ Agree □ Strongly agree

**B4. All childhood vaccines offered by the government programme in my community are beneficial**

□ Strongly disagree □ Disagree □ Neither agree nor disagree

□ Agree □ Strongly agree

**B5. New vaccines carry more risks than older vaccines**

□ Strongly disagree □ Disagree □ Neither agree nor disagree

□ Agree □ Strongly agree

**B6. The information I receive about vaccines from the vaccine program is reliable and trustworthy**

□ Strongly disagree □ Disagree □ Neither agree nor disagree

□ Agree □ Strongly agree

**B7. Getting vaccines is a good way to protect my child/children from disease**

□ Strongly disagree □ Disagree □ Neither agree nor disagree

□ Agree □ Strongly agree

**B8. Generally I do what my doctor or health care provider recommends about vaccines for my child/children**

□ Strongly disagree □ Disagree □ Neither agree nor disagree

□ Agree □ Strongly agree

**B9. I am concerned about serious adverse effects of vaccines**

□ Strongly disagree □ Disagree □ Neither agree nor disagree

□ Agree □ Strongly agree

**B10. My child/children does or do not need vaccines for diseases that are not common anymore**

□ Strongly disagree □ Disagree □ Neither agree nor disagree

□ Agree □ Strongly agree

**C. COVID-19 vaccine attitudes**

**C1. Will you vaccinate against COVID-19 for yourself?**

□ Yes (turn to C2) □ Not sure (turn to C3) □ No (turn to C3)

**C2. If “yes”, why? (Multiple choices)**

□ Risk of infection with COVID-19

□ COVID-19 is a serious disease

□ Protect the people around

□ COVID-19 vaccine is effective

□ Government publicity

□ Doctor's recommendation

□ Vaccine is free

□ Unit requirement

□ Personal needs (e.g. going abroad)

□ Others

**C3. If “no/not sure”, why? (Multiple choices)**

□ Concern about vaccine's safety or side effects

□ No risk of infection with COVID-19

□ Distrust vaccination clinics, hospitals or other medical professional institutions

□ Distrust vaccine manufacturers

□ Poor service quality of healthcare system

□ No professional gave me a detailed introduction to the vaccine

□ Conflict with belief

□ Cannot acquire the vaccine

□ Far from the vaccination clinic

□ I (or someone I know) had adverse experience in the previous vaccination

□ Want to immunize in a natural way rather than using a vaccine

□ Doubt the vaccine effectiveness

□ I get negative information about the vaccine in the media

□ I don't have time

□ Vaccination contraindications

□ Others
