# Supplementary material for: Changes in Parental Attitudes Toward COVID-19 Vaccination and Routine Childhood Vaccination During the COVID-19 Pandemic: Repeated Cross-sectional Survey Study
Source: JMIR Public Health Surveill. 2022 May 13;8(5):e33235. doi: 10.2196/33235 (PMC9109779; doi:10.2196/33235)
Supplement: Multimedia Appendix 2 [file publichealth_v8i5e33235_app2.docx]

**Table S1 Official websites of relevant health authorities**

| Health authority | URL |
| --- | --- |
| National Health Commission of the People’s Republic of China | <http://www.nhc.gov.cn/> |
| Jiangsu Commission of Health | <http://wjw.jiangsu.gov.cn/> |
| Jiangsu Provincial Center for Disease Control and Prevention | http://www.jshealth.com/ |
| Wuxi Commission of Health | http://wjw.wuxi.gov.cn/ |
| Wuxi Provincial Center for Disease Control and Prevention | http://wjw.wuxi.gov.cn/ztzl/wxsjbyfkzzx/sy/index.shtml |
