# Supplementary material for: Changes in Parental Attitudes Toward COVID-19 Vaccination and Routine Childhood Vaccination During the COVID-19 Pandemic: Repeated Cross-sectional Survey Study
Source: JMIR Public Health Surveill. 2022 May 13;8(5):e33235. doi: 10.2196/33235 (PMC9109779; doi:10.2196/33235)
Supplement: Multimedia Appendix 3 [file publichealth_v8i5e33235_app3.docx]

**Table S2 Participant’s vaccine hesitancy and COVID-19 vaccination willingness in three cross-section studies**

| Survey |  | Hesitant level | | COVID-19 vaccination willingness | | |
| --- | --- | --- | --- | --- | --- | --- |
|  |  | Low hesitant | High hesitant | Yes | Not sure | No |
| First survey | N | 2,656 | 225 | 1,709 | 1,078 | 94 |
|  | Unstandardized % | 92.2 | 7.8 | 59.3 | 37.4 | 3.3 |
| Second survey ^a^ | N | 867 | 171 | 716 | 271 | 51 |
|  | Unstandardized % | 83.5 | 16.5 | 69.0 | 26.1 | 4.9 |
|  | Standardized % | 84.9 | 15.1 | 64.6 | 29.3 | 6.1 |
| Third survey ^a^ | N | 1,116 | 67 | 1,102 | 75 | 6 |
|  | Unstandardized % | 94.3 | 5.7 | 93.2 | 6.3 | 0.5 |
|  | Standardized % | 94.5 | 5.5 | 92.0 | 7.2 | 0.8 |
| *P* ^b^ | | <.001 | | <.001 | | |
| *P* ^c^ | | <.001 | | <.001 | | |
| *P* ^d^ | | <.001 | | <.001 | | |
| *P* ^e^ | | .001 | | <.001 | | |

a: the two samples of the second and third survey were directly standardized by the age, gender and medical occupation distribution of the sample in the first survey to ensure the comparability of findings in three surveys, b: the comparisons among three surveys, c: the comparisons between first and second survey, d: the comparisons between second and third survey, e: the comparisons between first and third survey. The adjusted *P* using the Bonferroni method was 0.0167.
