# Supplementary material for: Changes in Parental Attitudes Toward COVID-19 Vaccination and Routine Childhood Vaccination During the COVID-19 Pandemic: Repeated Cross-sectional Survey Study
Source: JMIR Public Health Surveill. 2022 May 13;8(5):e33235. doi: 10.2196/33235 (PMC9109779; doi:10.2196/33235)
Supplement: Multimedia Appendix 4 [file publichealth_v8i5e33235_app4.docx]

**Figure S1 Vaccine hesitancy and COVID-19 vaccination willingness in three cross-section studies ^a^**

^a^ Samples from the second and third surveys were directly standardized according to age, gender, and medical occupation distribution of the sample from the first survey.


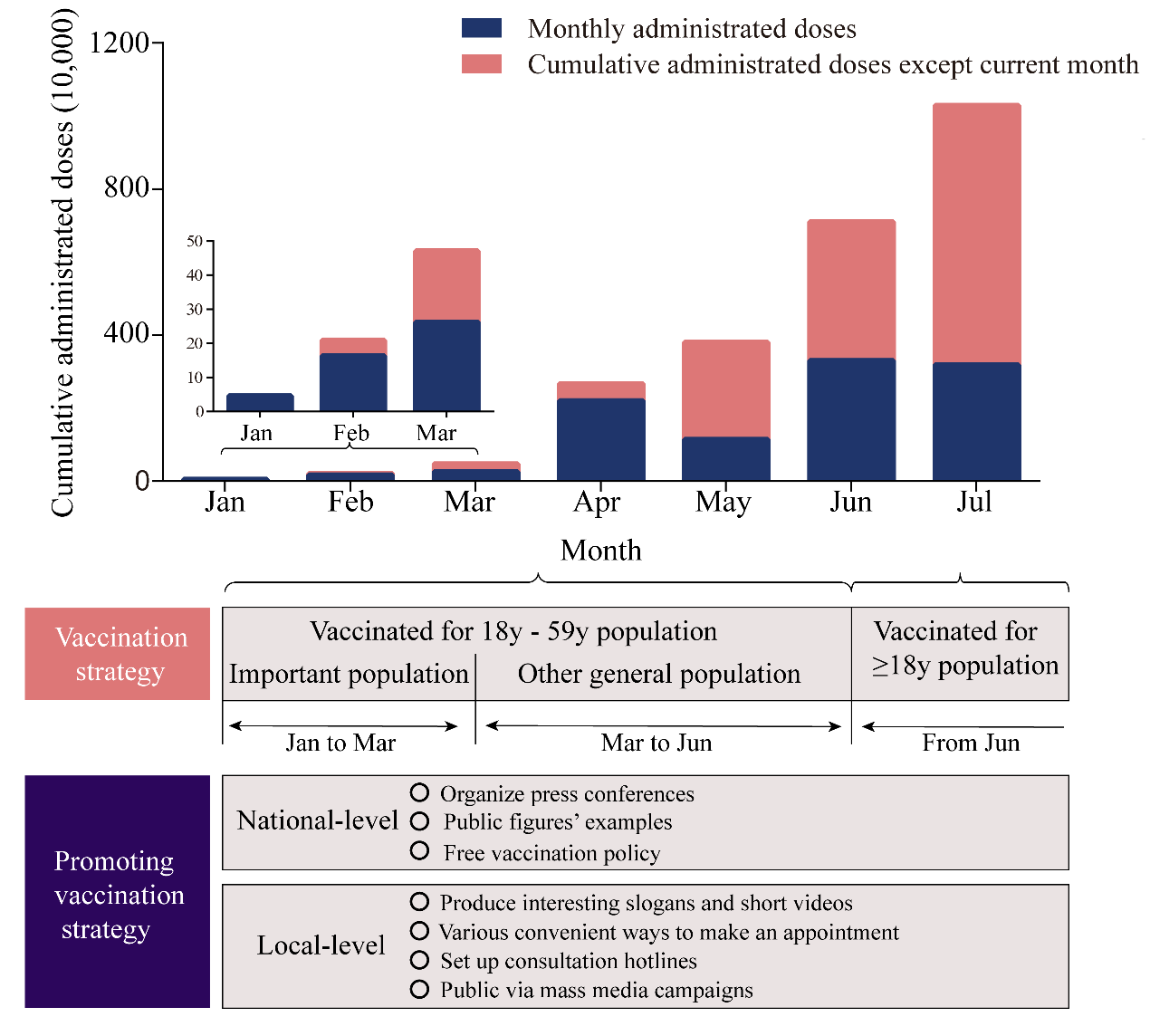


**Figure S2 Cumulative administrated COVID-19 vaccines in Wuxi city from January, 2021 ^a^**

^a^ Important populations included healthcare workers and workers in the following sectors: law enforcement and security, nursing home and social welfare institutes, community, energy, and food and transportation.


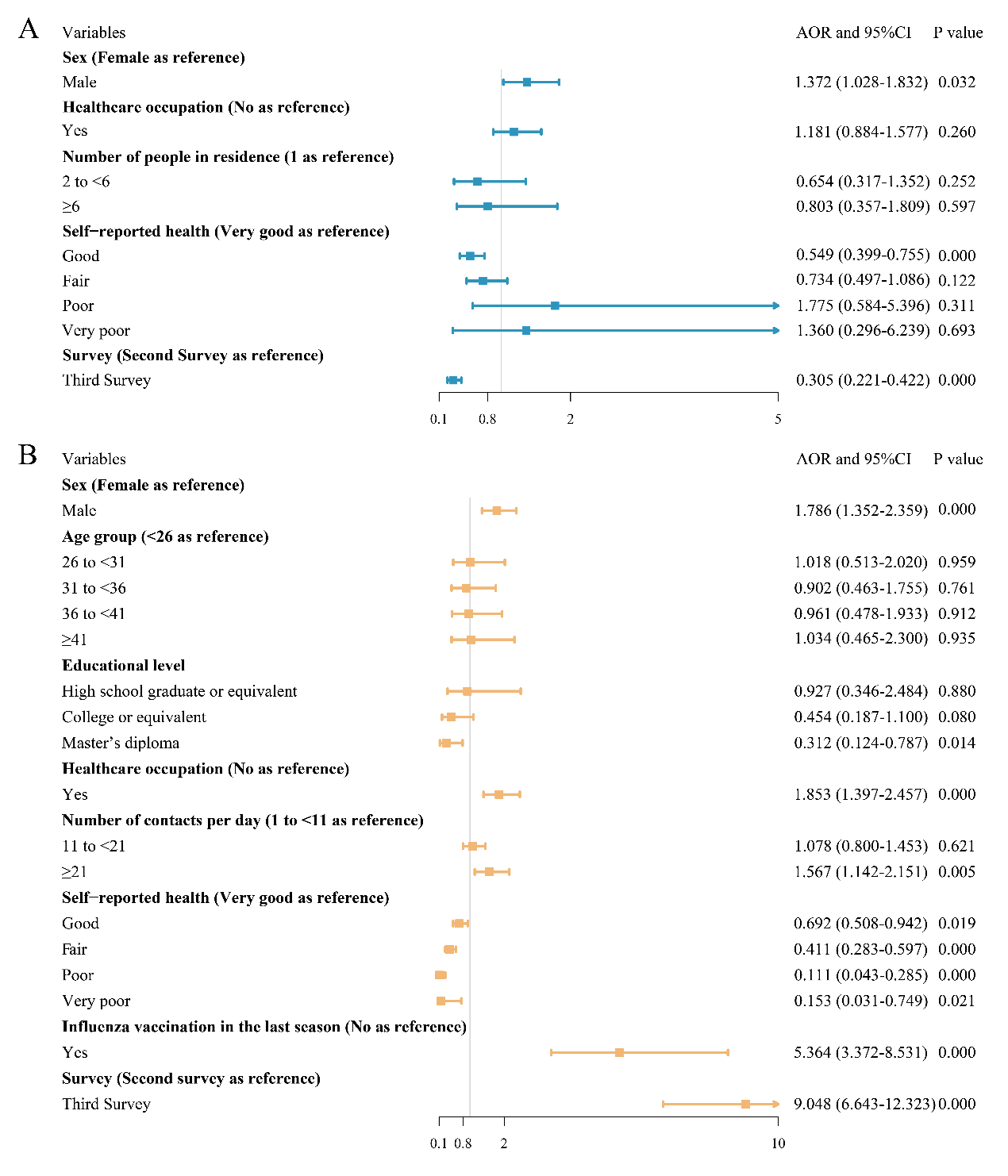


**Figure S3 Multivariable factors associated with parental vaccine hesitancy and** **COVID-19 vaccine willingness ^a^**

^a^ A: factors associated with parental vaccine hesitancy to routine childhood vaccine (“high-hesitant” as reference); B: factors associated with COVID-19 vaccine willingness (“yes” as reference); AOR: adjusted odds ratio; the vertical bar represents the invalid line (AOR = 1).


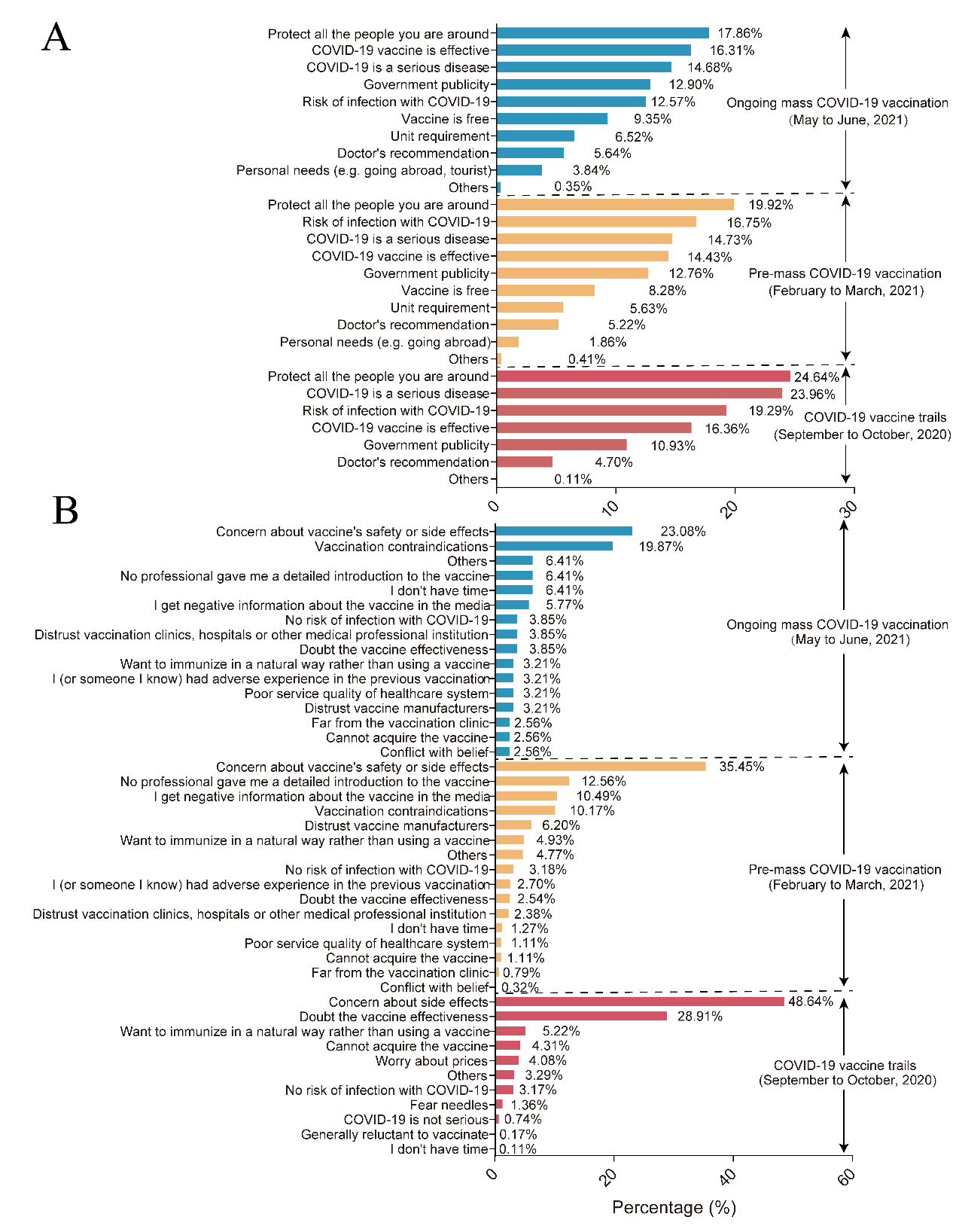


**Figure S4 Reasons for accepting and refusing vaccination against COVID-19 ^a^**

^a^ A: Reasons for accepting the COVID-19 vaccine for parents; B: Reasons for refusing the COVID-19 vaccine for parents.
